# Integrated Personal Health Records: Transformative Tools for Consumer-Centric Care

**DOI:** 10.1186/1472-6947-8-45

**Published:** 2008-10-06

**Authors:** Don Detmer, Meryl Bloomrosen, Brian Raymond, Paul Tang

**Affiliations:** 1 President and CEO, AMIA, Bethesda, Maryland, USA; 2 Vice President, AMIA, Bethesda, Maryland, USA; 3 Senior Policy Consultant, Kaiser Permanente Institute for Health Policy, Oakland, California, USA; 4 Chief Medical Information Officer, Palo Alto Medical Foundation, Palo Alto, California, USA

## Abstract

**Background:**

Integrated personal health records (PHRs) offer significant potential to stimulate transformational changes in health care delivery and self-care by patients. In 2006, an invitational roundtable sponsored by Kaiser Permanente Institute, the American Medical Informatics Association, and the Agency for Healthcare Research and Quality was held to identify the transformative potential of PHRs, as well as barriers to realizing this potential and a framework for action to move them closer to the health care mainstream. This paper highlights and builds on the insights shared during the roundtable.

**Discussion:**

While there is a spectrum of dominant PHR models, (standalone, tethered, integrated), the authors state that only the integrated model has true transformative potential to strengthen consumers' ability to manage their own health care. Integrated PHRs improve the quality, completeness, depth, and accessibility of health information provided by patients; enable facile communication between patients and providers; provide access to health knowledge for patients; ensure portability of medical records and other personal health information; and incorporate auto-population of content. Numerous factors impede widespread adoption of integrated PHRs: obstacles in the health care system/culture; issues of consumer confidence and trust; lack of technical standards for interoperability; lack of HIT infrastructure; the digital divide; uncertain value realization/ROI; and uncertain market demand. Recent efforts have led to progress on standards for integrated PHRs, and government agencies and private companies are offering different models to consumers, but substantial obstacles remain to be addressed. Immediate steps to advance integrated PHRs should include sharing existing knowledge and expanding knowledge about them, building on existing efforts, and continuing dialogue among public and private sector stakeholders.

**Summary:**

Integrated PHRs promote active, ongoing patient collaboration in care delivery and decision making. With some exceptions, however, the integrated PHR model is still a theoretical framework for consumer-centric health care. The authors pose questions that need to be answered so that the field can move forward to realize the potential of integrated PHRs. How can integrated PHRs be moved from concept to practical application? Would a coordinating body expedite this progress? How can existing initiatives and policy levers serve as catalysts to advance integrated PHRs?

## Background

Personal health records (PHRs) are consumer-centric tools that can strengthen consumers' ability to actively manage their own health and health care [1]. Although the capabilities of PHRs vary significantly in the current marketplace, they typically include provisions to capture information about an individual's diagnoses, medications, allergies, lab test results, immunization records, and other personal health information. Many PHRs also provide linkages to convenience tools (e.g., requesting appointments, requesting prescription renewals, asking billing questions) and communication tools to assist the patient in connecting with various health care professionals (e.g., physicians, nurses, pharmacists [2-5].

The concept of a PHR is not new [6]. What we now refer to as *personal health records (PHRs) *arose from low-technology solutions that individuals and families have used for many decades because they needed one place to record and access their complete medical history. Paper-based documents including clinical notes accumulated from various care providers, laboratory reports, and health histories are often compiled by health care consumers in envelopes, loose-leaf binders or shoe boxes. Generations of parents have used baby books to collect basic information on post-natal care, child development, medical consultations, and immunizations. Health information wallet cards are used by consumers to carry emergency medical contacts, blood type, and allergies. MedicAlert™ bracelets have become one of the most widespread ways to communicate basic health data to health professionals who might become involved with the patient needing emergency care.

Basic electronic personal health records emerged as people began collecting personal health information and entering it into computer-based, word processing templates or spreadsheet applications. These records are initiated and maintained by individuals, often to help them manage a chronic illness; they can include lifelong personal health information and can be used with or without the participation of health care providers.

As mass storage devices such as CD ROMs, smart cards, or USB flash drives became readily available they were utilized for maintaining personal health information. Early web-based PHRs include online emergency medical records that made manually-entered diagnoses, medications, and allergy information available to emergency room clinicians [7].

In today's parlance, a PHR typically refers to a computer-based record – either a standalone product (e.g., accessible on the Internet or on a USB drive) or one that is integrated with the provider's electronic health record (EHR). While the uptake of standalone PHRs has been slow, a growing number of patients actively use integrated PHRs [8].

Today, PHRs command attention on the national and international health policy landscape [9,10]. Recognizing that consumer engagement in health promotion and disease management is critical to quality improvement and health care cost containment strategies, [11] PHRs have been positioned as a tool to empower consumers to play a larger and more active role in wellness and self-care [12]. Health care leaders recognize that PHRs can integrate consumer and provider access to health information across the care continuum, including the home. Lessons learned from recent history (e.g., SARS, Hurricane Katrina) highlight the importance of portable personal health information in response and recovery efforts, the value of computer-based health records in the health care system, and the opportunity cost from the absence of these technologies.

In September 2006, the Kaiser Permanente Institute for Health Policy (Kaiser), the American Medical Informatics Association (AMIA), the Robert Wood Johnson Foundation (RWJF) and the Agency for Healthcare Research and Quality (AHRQ) convened a two-day invitational roundtable entitled "Personal Health Records and Electronic Health Records: Navigating the Intersection" with support from the Kaiser Permanente Foundation and the Robert Wood Johnson Foundation. The roundtable had three goals:

▪ Identify the transformative potential of integrated PHRs.

▪ Identify barriers to realizing this potential.

▪ Identify a framework for action to move integrated PHRs closer to the health care mainstream.

This paper highlights and builds on the issues and insights shared in the roundtable discussion. Time constraints did not allow roundtable participants to reach consensus on specific recommendations. Thus, the conclusions in this paper reflect the views of the authors only and do not necessarily represent the collective thinking of roundtable participants. These conclusions are offered as a contribution to the dialogue that is deepening our understanding of the transformative potential that can be realized when PHRs integrate with other health information systems and communication technologies.

Since the roundtable in September 2006, a range of PHR initiatives has advanced in planning and implementation; several of these are described later in this paper. Nevertheless, PHRs are still largely infant technologies and further dialogue, informed by research on pivotal issues, is needed to achieve steady progress towards integrated PHRs in this decade and the next.

## Discussion

### PHR models

Today, there is a spectrum of dominant PHR models [13,14]. *Standalone or free-standing PHRs *are often PC-based and require manual data entry to populate and update the record. Standalone PHRs help consumers organize and store medical data, can be accessed anytime and anywhere, and enable information sharing with providers. The most common free-standing PHRs are either paper-based, personal computer-based, or enabled by an Internet application. Some free-standing PHRs enable consumers to copy data onto convenient, portable storage devices. Some online variations of this model are offered by commercial organizations that derive revenue from sponsor advertising or data mining, while others charge a fee for maintaining information on a secure web page. The content of the free-standing PHR is typically created by and is under the physical control of the patient. Key limiting factors of the free-standing PHR are that manual data entry is typically required to populate and update the record [15] and practitioners may question the accuracy and completeness of self-reported/patient-entered information. And, like paper records, non-web-based PHRs (i.e., PC, mass storage devices) are vulnerable to destruction, theft, and loss.

*Integrated, interconnected, or networked web-based PHRs *can be populated with patient information from a variety of sources, including EHRs, insurance claims, pharmacy data, and home diagnostics and can provide consumers as well as providers with a more complete view of relevant health information. The consumer is an important contributor to the interconnected PHR content and is typically allowed to enter information into selected areas of the record. Integrated PHRs provide access for consumers to provider-based records; may eliminate manual re-entry of data; serve as a patient-provider communication channel; may reduce medical errors, eliminate duplication, and improve quality; enhance efficiency and convenience with online transaction tools; and promote a more comprehensive view of health status and health care activity. Some interconnected PHRs are offered in connection with services related to a specific health condition or disease and feature patient data integrated with personalized health advice and guidance [16].

Institution-specific, web-based PHRs (*tethered PHRs*) are a limited form of the integrated model that connect with a single provider-based EHR system or other institutional database, offering patients access to parts of their electronic health records via web portals. Additional functionality is often available with these systems, including the ability to e-mail medical providers, make follow-up appointments and renew prescriptions. These PHRs are a patient-facing extension of the clinician-controlled EHR, accessed via the Internet [17]. Patient data are under the physical control of the health care provider; however, in some systems, consumers can add to or annotate portions of the record.

Another approach receiving increased attention is the creation of PHRs using data derived from a patient's health insurance claims. While seeming to offer information to patients with minimal effort, the known, long-standing inadequacies of billing codes could result in as much confusion and misinformation as help. Further, these records could place significant burden on providers who will be forced to clarify or amend partial or erroneous diagnoses or related information.

### PHR functionality

Most standalone PHRs provide basic tools that help people collect, organize and store their health information [18]. These include medical history, medical and emergency contacts, outpatient and hospital visits, immunization tracking, insurance records, and health-related alerts and reminders. More advanced PHRs (particularly those with digitally-networked services) offer additional functions:

▪ Accessing medical records with capacity to offer amendments to add information (such as alternative treatments being pursued by the patient), or correct errors or incomplete information.

▪ Adding information of primary interest to patients rather than providers, such as patient-relevant decision support.

▪ Drug interaction checking (when a complete medication profile is available).

▪ Home monitoring with recording or tele-reporting of data to the record.

▪ Interactive health risk profiling and patient education resources.

▪ Patient-physician secure e-mail.

▪ Prevention and wellness reminders.

▪ Processing of claims and payment.

▪ Refilling of prescriptions.

▪ Retrieving of laboratory and other tests.

▪ Reviewing of insurance eligibility and benefits.

▪ Scheduling appointments.

### Transformative potential of integrated PHRs

Transformative health technologies are innovations that fundamentally change care, (including self-care), and care delivery in ways that add substantial value to individuals and society. When PHRs allow iterative communication between patients and providers, export data to and import data from other information systems, and transform clinical measurements and observations into meaningful and actionable information, fundamental changes in health care delivery and self-care by patients are possible. In this context, the value proposition of the integrated PHR far surpasses the value of the standalone PHR.

Thus, the transformative potential of integrated PHRs is realized through enhanced functionality. The data within an electronic PHR record alone are not sufficient to realize improvements that can be considered transformative. Significant value will be realized only when PHRs incorporate systems, tools, and other resources that leverage the data in the record and enable consumers to play a more active role in their health and health care. Some of these functionalities exist today; other applications are yet to be developed.

The major capabilities underlying integrated PHRs' potential as a transformative technology are outlined below.

▪ **Quality, Completeness, Depth, and Accessibility of Health Information**. Integrated PHRs improve the accuracy and completeness of health information provided by patients by capturing the data closer to the patient s experience and by capturing data generated by home monitoring. These data can be sent directly to health care providers when appropriate. When authorized, patient-generated data can be used for public health, research, [19] and performance measurement purposes.

▪ **Facile Communication**. Integrated PHRs permit both synchronous and asynchronous communication among patients, providers, and informal caregivers and provide tools for interactive decision-making.

▪ **Access to Health Knowledge**. Knowledge bases, self-care content, consensus guidelines, and best practices for both clinical and self-care can be integrated with PHRs through Internet connectivity.

▪ **Portability**. The true value of portable medical records and other personal health information lies in the ability of consumers to access all relevant sources of content from a single interface accessible anywhere, anytime through the Internet. Integrated PHRs hold this potential.

▪ **Auto-population**. Since many consumers will not have the skills, resources, or patience to compile their own health information, auto-population – the automatic insertion of reusable content – will be a key factor for long-term viability of PHRs [20]. Only through integration with other systems can PHRs systematically reuse information from cross-site data transfer among the disparate sources of content. The alternative (manual re-keying and transfer of information) is inefficient and error-prone. Auto-population of reusable content will increase the value of PHRs to consumers and providers by eliminating redundant data entry and ensuring more accurate, comprehensive, and timely content [21].

These capabilities will enable at least four advances in health care.

**First**, as integrated PHRs improve the availability of patient information at the point of care, interactions between patients and medical professionals will likely improve because practitioners will need to spend less time gathering patient history and be able to spend more time with patients probing deeper into concerns, questions, and clarification about their conditions [22-24]. Asynchronous Internet-based communication tools available in many integrated PHRs will improve patient-provider communication by avoiding "telephone tag"; enabling communication at the convenience of patients and providers; and automatically including patient-provider e-mail in the record.

**Second**, integrated PHRs enable electronic connectivity between clinical care managers and patients or their caregivers that can be leveraged to realize innovation in care management. The opportunities include capture of patient self-management information, data capture from home monitoring devices, links to peer support groups, and online coaching [25]. The likely payoff from online communication between providers and patients with chronic conditions will arise in improved treatment monitoring, more efficient use of time, potentially fewer office visits through substitution of online consultation for in-person visits, and improved continuity of care through common access to test results. Ultimately, integrated PHRs should enable comprehensive care that is 'virtually' accessible, continually available, and patient-centered [26].

**Third**, integrated PHRs should enable a shift in the health care locus of control to consumers by moving the control of health information from providers to patients or to a more "shared control" model consistent with the concepts of 'advanced medical home' or health home as discussed by the American College of Physicians (ACP) and others [27-30]. The American Academy of Pediatrics (AAP) introduced the medical home concept in 1967, initially referring to a central location for archiving a child s medical record. In its 2002 policy statement, the AAP expanded the medical home concept to include these operational characteristics: accessible, continuous, comprehensive, family-centered, coordinated, compassionate, and culturally effective care. The American Academy of Family Physicians (AAFP) and ACP have since developed their own models for improving patient care called the "medical home" or "advanced medical home." Empowering consumers to "own" and jointly manage the various sources of their health information increases the likelihood that providers will have a comprehensive view of patient information at the point of care.

Integrated PHRs will also support health knowledge promotion and lifestyle modification, and will provide benefits from the translation of clinical data into consumer-friendly health information. Further, they should stimulate patient-oriented decision support for managing chronic illnesses in tandem with clinicians. Creative approaches to fostering health education and lifestyle changes can be enabled with interactive, integrated PHR features that are not commonly available online (e.g., interactive health assessment, online support groups, reminders for preventive services).

**Fourth**, integrated PHRs should offer the following opportunities to reduce costs and improve health care delivery:

▪ Facilitate the sharing of patient and administrative information among otherwise closed health care systems and thereby reduce redundant transactions and tests.

▪ Promote more efficient use of time and facilitate substitution of online consultation for in-person visits.

▪ Enable home monitoring to remotely record patient data.

▪ Reduce the time practitioners spend gathering patient history.

▪ Enable the sharing of data with authorized patient proxies such as family members or other informal caregivers and allow authorized individuals to communicate with the health care team and stay abreast of the patient s welfare, irrespective of their geographic location.

As discussed later in this paper, formal evaluations are needed to quantify actual benefits as well as unanticipated, counter-intuitive effects of PHRs [31].

### Barriers to integrated PHRs

Development and widespread adoption of integrated PHRs will require understanding of and response to the factors that impede their adoption and potential contribution to the health system. These factors can be organized into the following major areas.

#### Health Care System Culture and Incentives

▪ **Balancing Physician and Patient Autonomy**. While the clinician-patient relationship has evolved significantly towards shared decision-making, the degree to which a historic paternalistic model persists may, depending on the patient s aspirations, create a barrier to collaborative care, information sharing, and joint decision making [32]. This is a particular concern when a patient's preferences (e.g., online communication, use of alternative sources of personal health information) are generally overridden or ignored by the clinician, or, alternatively, when the rare patient overuses the access feature and ignores the policies and procedures set out by the practice.

▪ **Scope of Work/Responsibilities**. Provider resistance to PHRs may stem from concerns about new processes and increased responsibilities associated with interacting with patients and using new health information technologies. Delbanco and Sands suggest that, "for doctors, at a time of disquiet, fatigue and bombardment by paper and electronic 'noise,' even if e-mail improves the quality of communications with patients it threatens to break the camel's back [33]." Given their many other responsibilities, practitioners may be unprepared to assume the role of "information broker"–helping patients look at health-related data from different sources and make informed decisions. Typically, patients are judicious in their communications and many, if not most clinician concerns are mitigated if they take the first step and start using such systems. Indeed, there is a reported decrease in 'phone-tag' and the capacity to carry out 'elective batched serial communications' by clinicians at the time of their choosing. For example, some clinicians report satisfaction from being able to leave the office, have dinner with their families, and then catch up on a few remaining patient e-mails from their home later in the evening since they can access the records via secure web portals.

▪ **Physician Compensation/Incentives**. Electronic patient-centered communication creates several categories of unfunded work for practitioners. The lack of compensation or other incentives for responding to patient e-mail, working with data from new sources, and facilitating informed/shared decision-making are key components of the problem. However, using standard evaluation and management (E&M) coding criteria, many electronic message threads can fulfill standard office visit reimbursement criteria (e.g., 99213).

▪ **Concerns (Real and Perceived) about Liability Risks**. Although most patients are not litigious, the widespread use of PHRs and other consumer-centric tools raises new potential areas of liability and risk for health care providers, such as the use of incomplete or inaccurate consumer-reported information, online clinician-patient communication, and privacy and security breaches [34].

#### Consumer Confidence and Trust

Perceived public concerns about security and confidentiality are a major hurdle to the electronic exchange of personal health information in light of the various media responses to breaches of health information systems and a very vocal and effective privacy advocacy community. Yet results from recent surveys suggest that although the public remains concerned about confidentiality and security issues, Americans are increasingly interested in the use of electronic health records to help improve their health care experiences and reduce costs.

A 2005 survey found that consumers rank the following issues as the absolute top priorities regarding the attributes of a health information exchange network [35].

▪ The identity of anyone using the system would be carefully confirmed to prevent any unauthorized access or any cases of mistaken identity.

▪ Individuals would be able to review who has had access to their personal health information.

▪ Only with an individual's permission could medical information be shared through a network.

▪ Employers and insurance companies would not have access to secure health information exchange networks.

A study of seniors in southern California found that while most respondents indicated that any PHR system must come from a trusted source, the majority of respondents expressed the view that privacy was not a high priority concern [36]. A 2006 Harris Interactive^® ^survey indicated that many U.S. adults are generally satisfied with how their personal health information is used. A majority agreed that increased use of computers to record and share patient medical records can be accomplished without jeopardizing patient privacy rights [37]. Another 2006 survey sponsored by the Markle Foundation found that two-thirds of the public is interested in accessing their own personal health information electronically. Eighty percent of those surveyed remain concerned about identify theft, fraud, or the possibility of their information becoming available to marketers [38]. A 2007 national survey commissioned by the Institute of Medicine found that only 1% of respondents would be comfortable having their health and medical information freely used by researchers without their consent [39]. As discussed below, these results point to the need for additional research grounded in actual practice.

#### Lack of Technical Standards for Interoperability

Interoperability refers to the ability of systems to interact with one another and exchange data according to a prescribed method in order to achieve predictable results. The immaturity and slow diffusion of standards for interoperability and data portability are key barriers to the integration and exchange of structured data among PHRs and the range of relevant entities that provide and finance health care. ISO TC 215 WG1 (Health Informatics) has published a technical report on personal health records and the need for standards. The report notes that growing interest around the world in PHRs and their potential standardization is driven by convergent interests among the consumer electronic industry, the established medical devices industry, health service providers and citizens [40]. Several standards necessary for integrated PHRs are described below.

▪ **Data Interchange Standards**. The codification of data, the structure and format of messages, and the health care vocabularies that promote comparable and consistent information.

▪ **Common Data Set/Minimum Data Set**. A core data set to ensure that a minimum amount of data is available to consumers and providers for self-care and clinical encounters (e.g., patient and provider identification, insurance information, allergies, medications, vital signs, diagnoses, recent procedures). A default set of fields will likewise have implications for PHR developers, EHR developers, and custodians of professionally-sourced health data (e.g., health plans, pharmacy benefits managers, and retail pharmacies) [41].

▪ **Consumer Terminologies**. Augmentation of formal health care vocabularies with lay vernacular.

▪ **Authentication Processes**. Entity and individual authentication to protect against unauthorized disclosure of personal health information.

▪ **Identification Processes**. Positive patient identification processes and systems to facilitate networking of patient information, to avoid breaches of confidentiality, and to avoid preventable medical errors [42-45].

▪ **Security Standards**. Administrative procedures, physical safeguards, technical data security services and technical security mechanisms.

▪ **Data Integrity Processes**. Security mechanisms to ensure that data has not been altered or corrupted, either accidentally or intentionally in an unauthorized manner.

▪ **Privacy Standards**. Outlining of specific rights for individuals and obligations for organizations holding PHR data regarding protected health information [46]. This may include developing privacy options for those individuals whose concerns for privacy are of less importance to them than their interest in sharing their person-specific health information for medical research or other socially beneficial uses.

▪ **Certification**. Application of objective criteria against which health information technology products can be evaluated to ensure compliance with data interchange standards.

#### Lack of HIT Infrastructure

▪ **High Enterprise Cost of Data Integration**. The integration of health information from disparate sources is a daunting task fraught with considerable obstacles. Today, there is a general lack of affordable, out-of-the-box integration solutions to handle the cleansing, formatting, and mapping of health information from multiple sources into a coherent and meaningful format. The costs associated with inter-institutional connectivity exceed the IT infrastructure budgets for most health care organizations, requiring the allotment of highly-skilled, in-house resources or large expenditures for consulting services.

▪ **No Mediating Structure**. Initiatives are underway in most states to develop networks of sufficient size and scale to serve as the infrastructure to support the exchange of health information among relevant stakeholders (e.g., patient identification, record location, authentication, access controls). Collaborative initiatives known as Regional Health Information Organizations (RHIOs) involving hospitals, physician practices, laboratories, pharmacies, and other organizations are being explored as a possible model for health information exchange at a regional level. Likewise, SubNetwork Organizations (SNOs) are a model for health information exchange sponsored by non-geographic communities of interest that represent populations defined by common values, needs, concerns or organizational affiliation (e.g., national disease organizations, consumer interest groups). RHIOs and SNOs, however, are still largely conceptual; only a small number of demonstration projects have advanced beyond planning into implementation [47].

▪ **Limited Online Services Offered**. Nearly half of respondents to a survey of U.S. health care professionals indicated that their organization does not offer patients the ability to access online services, such as prescription refill. Only 20 percent indicate that their organization offers patients portal access to online services [48].

#### Equity and Usability: The Digital Divide

The continuing digital divide between those with and those without the ability to effectively use digital information technology is an obstacle to the promotion and use of integrated PHRs. Drivers of the digital divide include:

▪ **Racial and Socio-Economic Disparity Gap**. The difference in computer and Internet access to health care information is largely a function of race, education and socioeconomic status [49]. For example, African-Americans and Latinos are substantially less likely to have a home computer and use the Internet than are white non-Latinos [50]. There is strong evidence, however, that e-health systems will be used extensively and have a positive impact on low-income patients with access to such technology [51].

▪ **Health Illiteracy**. A study of information technology use and literacy found that nearly one of two U.S. adults has difficulty understanding information necessary to make basic health decisions [52].

▪ **Special Needs**. Special adaptive tools (e.g., alternative computer input devices) may be required for individuals with visual impairment or physical limitations.

▪ **Lack of Financial Resources**. Health care safety net agencies are especially challenged by a lack of funding for technical infrastructure and expertise to support health IT services [53].

#### Value Realization/ROI

Health IT investments usually require justification based on quantifiable benefits in terms of avoided cost, improved efficiency or increased revenue. The health IT business case needs to take into consideration the one-time infrastructure and labor costs for implementation, as well as ongoing system support costs. Integrated PHRs are no exception to cost benefit justification, but a variety of factors have made the integrated PHR business case difficult to ascertain.

▪ There is a lack of empirical evidence in health care and informatics literature to quantify the PHR value proposition. While many of the perceived PHR benefits accrue to consumers, it is not clear that they are willing to pay or subsidize the cost of electronic health records. Although surveys consistently show substantial numbers of consumers indicating their willingness to pay for integrated PHRs, [54-56] this has not yet been demonstrated in practice.

▪ Within the current business model, savings under non-capitated reimbursement arrangements tend to accrue to payers rather than the entity that invests in the technology.

▪ Benefits such as patient satisfaction, improved communication, and consumer engagement are not easily quantifiable.

#### Uncertain Market Demand

Like other forms of electronic health records, integrated PHRs offer both significant potential benefits for users and a high degree of risk for potential investors. The uncertain market demand arises from a host of factors.

▪ Absence of information about whether there is adequate patient knowledge about as well as demand for the integrated PHR or its applications.

▪ Absence of information about whether there is adequate knowledge as well as demand by other stakeholders.

▪ Absence of information about whether there is adequate value for each stakeholder.

▪ Concerns about who should pay and how much they should pay.

▪ Absence of aligned incentives in the majority of the U.S., given the fragmented health care delivery system.

▪ Concerns about strong incentives for some stakeholders to develop proprietary systems with limited functionalities.

▪ Absence of information about the sustained value of integrated PHRs.

▪ Concerns about the need for a critical mass of data sources and level of integration.

▪ Absence of information on how workforce and work processes will change.

In combination, these factors reinforce the need for public sector and philanthropic investment to increase the information needed to allow the market to assess the merits of integrated PHRs.

### Recent progress toward integrated PHRs

In spite of the significant obstacles to achieving the potential of integrated personal health records, there are promising signs of progress. Taken together, they point toward a potential national model for maintaining, populating, and sharing health information in PHRs [57].

#### A Common Framework for Networked Personal Health Information

In December 2006, The Connecting for Health Personal Health Technology Council released *A Common Framework for Networked Personal Health Information *that identified a model for integrating consumer-centric health IT applications across the health care delivery system [58]. The Framework builds on the fundamental design elements of earlier versions of the Connecting for Health Common Framework model and describes a networked environment in which consumers could securely exchange their personal health information. The Framework is a federated, decentralized network of networks that permits consumers and other stakeholders to direct "information from disparate data sources into electronic health records, including PHRs." However, currently, nearly all existing PHR implementations are centralized; there are no implementation examples of the federated PHR model as described in the Common Framework.

#### Emerging PHR Interoperability Standards

Several important milestones have been reached recently towards the goal of a higher degree of data and information exchange among providers and consumers.

▪ The Healthcare Information Technology Standards Panel (HITSP) [59] recommended and DHHS Secretary Mike Leavitt accepted a set of Consumer Empowerment Interoperability Specifications for consumers to exchange data with their providers. They include use cases and recommended standards for the basic functions of medication history and registration summary, as well as standards for permission access rights and informed consent for exchange of health information.

▪ The Health Level Seven (HL7) Continuity of Care Document (CCD) reflects multiple years of effort by clinical and health informatics stakeholders to harmonize two sets of separately developed, but complementary standards for clinical document architecture: the American Society for Testing and Materials (ASTM) Continuity of Care Record (CCR) and the HL7's Clinical Document Architecture (CDA) [60]. The CCD can facilitate sharing of a consumer's most relevant administrative and clinical information, including health status, health care treatment, insurance information, advance directives, and caregivers. On November 5, 2007, HL7 announced the release of a ballot to approve its Personal Health Record System Functional Model (PHR-S FM) as a Draft Standard for Trial Use (DSTU) [61].

▪ The Portable Document Format (PDF) created by Adobe Systems for desktop publishing is an open standard that is being adopted for health care information exchange by voluntary standards development organizations and other industry leaders. A new PDF/H (PDF-Healthcare) has been proposed as a portable, secure, and universal health care data exchange container for personal health records and electronic health records [62].

#### U.S. Federal Government Programs

The Centers for Medicare & Medicaid Services (CMS) and the Department of Veterans Affairs (VA) are carrying out major new pilots to test various aspects of personal health records with their constituencies. In June 2007, CMS announced a new project (Registration Summary/Medication History PHR)  expanding its efforts to encourage Medicare beneficiaries to take advantage of Internet-based tools to track their health care services and to provide them with resources to better communicate with their providers. This pilot program is intended to enable certain beneficiaries to use a PHR provided through participating health plans, accessible through .

CMS launched the program in conjunction with four health plans to test the use of their PHRs. The plans are HIP USA, Humana, Kaiser Permanente, and the University of Pittsburgh Medical Center. Each plan has a unique PHR tool that will be accessible to beneficiaries. The availability of different tools is expected to provide valuable information to CMS on the various features offered, including which are most popular and useful to the individual [63].

This CMS study is part of a larger PHR action plan, which describes a number of ways that CMS can help promote the growth of PHRs and ensure that beneficiaries have private and secure access to their own health care information. CMS' action plan supports the activities being undertaken by the Office of the National Coordinator (ONC), the DHHS Office of the Assistant Secretary for Planning and Evaluation (ASPE), AHRQ, and the American Health Information Community (AHIC).

The VA is testing My HealtheVet Pilot,  a prototype developed to demonstrate that the agency can provide veterans with a safe, secure, and private electronic copy of their own VA health information through a web environment. Pilot registrants can obtain copies of key portions of their electronic health records; add structured medical data in the "self-entered" section of the record; track personal health metrics (blood pressure, weight, etc.); access health education materials; and grant access to their health information to family members and VA and non-VA health providers.

#### New Models for Health Information Storage and Exchange

Although the market for consumer-oriented health data warehousing is still in its infancy, there are several emerging models that create new opportunities for consumers to control and share their health information. For example, a consortium of major national employers announced plans to sponsor Dossia, a non-profit, independent data warehouse in which their employees can maintain lifelong personal health information [64]. Microsoft  won the race among large technology companies to launch online health information repositories that allow consumers to import, store, and share health records from various sources [65]. With Google Health , which debuted in May 2008, users can create a personal health profile; import medical records and prescription history from healthcare providers through secure linkages; check new medications for drug interactions or allergies; refill prescriptions; ask for a second opinion; get personalized health information; and search for doctors and other medical services.

One increasingly popular implementation approach to integrated PHRs is the Health Record Bank (HRB), defined as "an independent organization that provides a secure electronic repository for storing and maintaining an individual's lifetime health and medical records from multiple sources and assuring that the individual always has complete control over who accesses their information." . The focus is on the objective service of maintaining individual EHRs, much like financial banks maintain and manage financial assets. Legislation would create multiple, competing, regulated independent HRBs, owned neither by healthcare providers nor by payers or government agencies [66]. Through the ePHR, (the equivalent of a bank's individual or joint personal account), the patient can control his or her own data, keep a complete health record, and make any or all of the data accessible to providers, as well as other authorized users [67].

Revolution Health , a consumer-centric health company developed by AOL co-founder Steve Case, features consumer-controlled health record "banks" bundled with health education, social networking and health expense management tools. The common themes in these models are that medical records are centrally located and accessible using a secure Internet site and that the consumer controls who can make "deposits" to and "withdrawals" from their account. These models establish a consumer-selected custodian of personal health information.

#### Examples of Personal Health Record Initiatives Outside of the U.S

Health systems in other countries are gaining experience working with a variety of personal health record programs.

▪ **Andalucia, Spain**. DIRAYA (Arabic for knowledge) is an integrated, citizen-centered health solution that maintains a unified EHR based on a number of interoperable elements. It is based on 4 principles: a single health record for each person; unified access to all services; structuring (coding) of all relevant information; and system development by practitioners and providers. As the development of DIRAYA got underway, a fifth principle was adopted: "customer precedence" in which patients are not considered to be customers or clients, but rather owners. In 2007, DIRAYA had been implemented in 88% of the primary healthcare centres which cover 79% of the Andalucia population [68].

▪ **Scotland**. NHS Scotland s Emergency Care Summary enables clinicians in hospital accident and emergency departments to access, with patient consent, crucial medical information on prescribed medications and allergies 24 hours a day. The program now securely holds over 5 million patient records, and has been accessed by health professionals more than 1 million times. Patients can choose to opt out of the program at any time [69].

▪ **Denmark**. The Health Portal  provides access for health professionals to patient data in the laboratory systems and in local electronic patient records, following patient consent. It enables patients to request appointments and renew prescriptions and enables e-mail consultation between patients and physicians [70].

### Next steps for advancing integrated PHRs

The PHR universe is an evolving space, with much work remaining to be completed on multiple fronts to advance integrated PHRs. As mentioned in the Background section of this paper, time constraints did not permit roundtable participants to develop a comprehensive list of needed actions. Their discussions did, however, suggest three key areas where private and public sector organizations can focus attention and resources to help advance integrated PHRs in the short term.

#### Share Existing Knowledge about Integrated PHRs

Compilation of structured, easily accessible information about the benefits gained from existing integrated PHRs and the best practices for integrated PHR development and implementation would be an important step towards supporting organizations interested in pursuing integrated PHRs as a clinical and business strategy. This could take the form of a compendium that highlights standards of practices in PHR deployment, administration, and use. Issues addressed in the compendium could include authentication policies and procedures, e-mail response time for patient messages, communication policies regarding abnormal lab results, longitudinal record modeling, and informed consent and perspectives on consumer rights. Work in this area could build on the AMIA *Guidelines for the Use of Clinic-Patient Electronic Mail *[71] and should complement the efforts of the Markle Foundation's Connecting for Health initiative.

#### Expand Knowledge about Integrated PHRs

A focused research agenda is needed to inform the development and implementation of integrated PHR systems, guide education about these systems, and support the development of principles of responsibility for stakeholders. For example, while much discussion has addressed the potential of personal health records, there are relatively few rigorous quantitative studies that document their impact. The agenda should be used to inform the work of public research agencies and funders such as the National Institutes of Health (NIH), CMS, ONC, and AHRQ. This research agenda should also be shared with similar advisory groups of other nations.

The research agenda should address:

▪ Evolving desired functionalities for integrated PHRs including studies that solicit future functionalities from the perspectives of patients, special populations, payers, providers, regulators, patient advocacy groups, etc.

▪ Development and refinement of integrated PHR models for health communications and care, and identification of the applications and devices that hold the greatest transformative potential.

▪ Impact studies on the effectiveness of PHRs through a systematic review of business cases and clinical use cases, and on the impact of PHRs on individual health and their potential for proactive prevention and disease prediction.

▪ Evaluation of models of care delivery that are integrated with PHRs and PHR systems.

▪ Liability issues and other legal barriers that confront PHR implementers.

▪ Implications of integrated PHRs' use of multisource, heterogeneous and context-aware information for privacy protection, security and semantic interoperability.

▪ Use of informed consent with the integrated PHR as a process for individuals to authorize the exchange of personal health information for various purposes (e.g., health data reuse for public health, research purposes).

▪ Needs of special populations including rural, minority, central city poor, physically handicapped, and non-English speaking persons.

In the short term there is a need to obtain additional sound, objective, and credible information about consumers' views of the value of integrated PHRs and desired PHR functionalities [72,73]. For example, Project HealthDesign (PHD), the Robert Wood Johnson (RWJ) initiative to support creation of a new generation of personal health record (PHR) systems, released an advanced draft of a set of functional requirements which the program believes will be common to most PHR applications. This information is particularly needed since technological and societal forces are shifting.

An example of a survey addressing this need was commissioned by the Markle Foundation and conducted in May 2008 [74]. A total of 1,580 American adults nationwide were asked about their views on the value of individually controlled electronic PHRs and privacy considerations related to these PHRs. The survey was the first to be conducted on a national scale that explored consumer perceptions about PHRs after the entrance of Google, Intuit, Microsoft, Revolution Health and WebMD into this marketplace and to measure perceptions of the importance of privacy practices in decisions to use such services.

The 2008 Markle-commissioned survey found that only 2.7 percent adults have an electronic PHR (representing about 6.1 million persons). In the future, such a survey should include a sufficiently large sample of those patients who get care through the use of integrated PHRs to determine their views of PHRs' value (ability to manage chronic illness, implications for lifestyle changes and lifelong care education) and concerns about data security and privacy issues.

#### Identify and Build upon Existing Efforts that Relate to Integrated PHRs

A range of existing activities within the health information technology domain do or could support development and use of integrated PHRs. This support should be made explicit through planning and resource allocation. These activities include, but are not limited to the following:

▪ **Standards development organizations **advancing interoperability standards that promote integration of PHRs with EHRs by developing PHR data standards that are consistent with EHR data standards.

▪ **EHR vendors **supporting integrated PHRs by agreeing upon common PHR standards for electronic data importation and exportation and other core functionality by 2009, and supporting integrated PHRs by including PHR functionality in their products by 2009.

▪ **Certification Commission for Healthcare Information Technology (CCHIT) **certifying security and confidentiality standards for integrated PHRs as soon as possible and certifying integrated PHR/EHR systems by 2009. This effort can build on minimum standards development underway relating to data elements and a platform of basic functions.

▪ **National entities broadly promoting EHRs **and explicitly addressing integrated PHRs. AHIC, the National Committee on Vital and Health Statistics (NCVHS), CCHIT and other relevant agencies or regulatory bodies dealing with electronic health records should acknowledge that PHRs are an integral component of health care communications and record-keeping by including appropriate policy, standards, demonstration projects, education, training and research efforts in their work agendas. And while several of these organizations are currently addressing PHR-related issues, moving PHRs toward a higher level of interconnectivity should be earmarked for priority action. Work plans of these entities should reflect this dimension in 2009 at the latest.

▪ **RHIOs and RHIO initiatives **incorporating PHR integration into their planning and development efforts. RHIOs are potential enablers of integrated PHRs because of their ability to serve as focal points for authentication, authorization and data exchange among PHR and EHR stakeholders.

## Summary

Two principal dimensions of consumer engagement in health care are at the heart of the PHR opportunity: consumer access, and to a varying extent, control over consumer health information; and active, ongoing patient collaboration in care delivery and health care decision making, including the capacity to evaluate their own health status and progress over time. The integrated PHR model asks consumers to be willing to engage with their providers in an integrated, web-based, secure (but not totally foolproof) record and communication system.

With some exceptions, however, the integrated PHR model is still a theoretical framework for consumer-centric health care. The integrated PHR framework will require a secure, patient-controlled, lifelong record that aggregates data from all relevant sources and is accessible at any time, any place. Transparency, including the consumer's ability to determine who has accessed or modified any part of their record, is an essential part of the consumer-centric framework. And finally, the framework must address the issues of data exchange with other information systems and health professionals [75].

These attributes suggest an interoperable network for new channels of communication and care management. And they point toward a new tool that is clearly broader than the legal record of any provider. As traditional roles and relationships between consumers and different parts of the health care delivery and financing system are fundamentally altered by a more consumer-centric framework, stakeholders may realize a variety of new benefits from interaction with PHRs. For example, Project Health Design, the RWJ initiative mentioned above, is stimulating PHR innovation through grants to design and test a suite of consumer-centric health applications [76].

Several key questions are clear after exploring the opportunities and challenges to creating an environment in which to realize the full potential of integrated PHRs.

▪ How do we get from integrated PHR concepts to widespread practical application?

▪ Privacy and security concerns present a two fold dilemma: How can unbiased public privacy surveys [77] that accurately measure consumers true preferences and concerns be funded and disseminated? How should integrated PHR advocates confront the actual, rather than perceived, risks to the privacy, confidentiality, and security of personal health information?

▪ To what extent would a coordinating body or structure expedite progress towards integrated PHRs through communication, coordination, priority setting, and pooling of resources?

▪ How can existing initiatives and policy levers serve as catalysts to advance integrated PHRs?

Further dialogue among public and private sector stakeholders is needed to determine how to approach the complex issues surrounding integrated PHRs.

## Competing interests

Brian Raymond has no competing interests. Meryl Bloomrosen has no competing interests. Don Detmer has no competing interests. Paul Tang has previously served on Google's Google Health Advisory Council and CapMed's Medical Advisory Board. However, he has no equity interests in either organization. Paul Tang currently serves as co-chair of the Certification Commission for Healthcare Information Technology (CCHIT) Personal Health Record (PHR) Advisory Task Force.

## Authors' contributions

MB, DD, BR, and PT have each made substantial contributions in drafting, writing and revising the multiple versions of the manuscript. FT and ES worked as consultants to AMIA and helped edit the document.

## Pre-publication history

The pre-publication history for this paper can be accessed here:



## References

[B1] American Medical Informatics Association and the American Health Information Management Association (2007). Joint Position Statement for Consumers of Health Care: The Value of Personal Health Records. http://www.amia.org/files/ahima-amiaphrstatement.pdf.

[B2] Sprague L (2006). Personal Health Records: The People's Choice?. National Health Policy Forum Issue Brief 820.

[B3] Sittig D (2002). Personal health records on the internet: a snapshot of the pioneers at the end of the 20th Century. Int J Med Inform.

[B4] Tang PC, Ash JS, Bates DW, Overhage JM, Sands DZ (2006). Personal Health Records: Definition, Benefits, and Strategies for Overcoming Barriers to Adoption. J Am Med Inform Assoc.

[B5] Masys D, Baker D, Butros A, Cowles KE (2002). Giving patients access to their medical records via the internet: the PCASSO experience. J Am Med Inform Assoc.

[B6] National Committee on Vital and Health Statistics (2006). Personal Health Records and Personal Health Record Systems: A Report and Recommendations.

[B7] Gearon CJ (2007). Perspectives on the Future of Personal Health Records. California. Health Care Foundation.

[B8] Altarum Institute Environmental Scan of the Personal Health Record (PHR) Market DRAFT.

[B9] Smith J (2000). Sea change? The personal health record and consumerism. Healthcare Information Management and Communications Canada.

[B10] Steinbrook R (2008). Personally Controlled Online Health Data The Next Big Thing in Medical Care?. N Engl J Med.

[B11] Pagliari C, Detmer DE, Singleton P Potential of personal health records. BMJ.

[B12] Hassol A, Walker JM, Kidder D, Rokita K, Young D, Pierdon S, Deitz D, Kuck S, Ortiz E (2004). Patient experiences and attitudes about access to a patient electronic health care record and linked web messaging. J Am Med Inform Assoc.

[B13] Kaiser Permanente Institute for Health Policy (2007). Realizing the Transformative Potential of Personal Health Records. Focus.

[B14] Cronin C (2006). Personal Health Records: An Overview of What Is Available To the Public. AARP.

[B15] Endsley S, Kibbe DC, Linares A, Colorafi K (2006). An introduction to personal health records. Fam Pract Manag.

[B16] Waegemann CP Testimony to National Committee on Vital and Health Statistics Regarding Personal Health Records.

[B17] Tang PC, Ash JS, Bates DW, Overhage JM, Sands DZ (2006). Personal Health Records: Definition, Benefits, and Strategies for Overcoming Barriers to Adoption. J Am Med Inform Assoc.

[B18] Kim MI, Johnson KB (2002). Personal health records: evaluation of functionality and utility. J Am Med Inform Assoc.

[B19] Mandl KD, Kohane IS Tectonic shifts in the health information economy. N Engl J Med.

[B20] Bauer J (2006). Creating a seamless IT enterprise: the rest of the story. J Healthc Inf Manag.

[B21] Bieliková M, Moravcik M, Manolis W, Angelides MC, Phivos M (2008). Modeling the Reusable Content of Adaptive Web-Based Applications Using an Ontology. Advances in Semantic Media Adaptation and Personalization 2008 Studies in Computational Intelligence.

[B22] Wang M, Lau C, Matsen FA, Kim Y Patient-centered health record linked to a referral service. http://depts.washington.edu/pettt/papers/Referrals.pdf.

[B23] Luo J (2006). Personal health records. Prim psychiatry.

[B24] Kim E, Wang M, Lau C, Kim Y (2004). Application and Evaluation of Personal Health Information Management System. Engineering in Medicine and Biology Society 2004 IEMBS 04 26th Annual International Conference of the IEEE.

[B25] Joslyn JS (2001). Healthcare e-commerce: connecting with patients. J Healthc Inf Manag.

[B26] Tang PC, Lansky D (2005). The missing link: bridging the patient-provider health information gap. Health Aff (Millwood).

[B27] The American College of Physicians (2006). Personal Health Records Policy Statements. http://www.acponline.org/advocacy/where_we_stand/health_information_technology/phr.pdf.

[B28] American College of Physicians The Advanced Medical Home: Patient-Centered, Medical Home Overview. Policy Monograph.

[B29] American Academy of Family Physicians, American Academy of Pediatrics, American College of Physicians, American Osteopathic Association (2007). Joint Principles of the Patient-Centered Medical Home. http://www.medicalhomeinfo.org/.

[B30] The American College of Physicians (2006). Personal Health Records Policy Statements. http://www.acponline.org/advocacy/where_we_stand/health_information_technology/phr.pdf.

[B31] Halamka JD, Mandl KD, Tang PC (2008). Early experiences with personal health records. J Am Med Inform Assoc.

[B32] Kohn L, Corrigan JM, Donaldson MS, Institute of Medicine, Committee on Quality of Health Care in America (2000). To Err is Human: Building A Safer Health System.

[B33] Delbanco T, Sands DZ Electrons in flight–e-mail between doctors and patients. N Engl J Med.

[B34] Pagliari C, Detmer DE, Singleton P Potential of personal health records. BMJ.

[B35] Markle Foundation (2005). Attitudes of Americans Regarding Personal Health Records and Nationwide Electronic Health Information Exchange. http://www.phrconference.org/2005/assets/research_release_101105.pdf.

[B36] Lafky DB Putting the consumer in the driver's seat: a user-driven approach to PHR design. Testimony before the American Health Information Committee, Consumer Empowerment Workgroup.

[B37] Harris Interactive® Many U.S. Adults are Satisfied with Use of Their Personal Health Information. The Harris Poll #27.

[B38] Lake Research Partners and American Viewpoint (2006). Survey Finds Americans Want Electronic Personal Health Information to Improve Own Health Care. http://www.phrconference.org/index.php.

[B39] Westin AF (2007). How the Public Views Privacy and Health Research. Institute of Medicine.

[B40] Rowlands D (2007). Report on ISO TC215 (Health Informatics) standards development meetings, Montreal. Standards Australia s Health Informatics Technical Committee (IT-014).

[B41] Markle Foundation (2004). Working Group on Policies for Electronic Information Sharing Between Doctors and Patients: Final Report: Connecting Americans To Their Healthcare. http://www.connectingforhealth.org/resources/wg_eis_final_report_0704.pdf.

[B42] Lee AC, Leung M, So KT (2005). Managing patients with identical names in the same ward. Int J Health Care Qual Assur Inc Leadersh Health Serv.

[B43] Gray JE, Suresh G, Ursprung R, Edwards WH, Nickerson J, Shiono PH, Plsek P, Goldmann DA, Horbar J (2006). Patient misidentification in the neonatal intensive care unit: quantification of risk. Pediatrics.

[B44] Bittle MJ, Charache P, Wassilchalk DM (2007). Registration-associated patient misidentification in an academic medical center: causes and corrections. Jt Comm J Qual Patient Saf.

[B45] McDonald CJ (2006). Computerization can create safety hazards: a bar-coding near miss. Ann Intern Med.

[B46] Markle Foundation (2004). Working Group on Policies for Electronic Information Sharing Between Doctors and Patients: Final Report: Connecting Americans To Their Healthcare. http://www.connectingforhealth.org/resources/wg_eis_final_report_0704.pdf.

[B47] Simons WW, Halamka JD, Kohane IS, Nigrin D, Finstein N, Mandl KD (2006). Integration of the personally controlled electronic medical record into regional inter-regional data exchanges: a national demonstration. AMIA Annual Symposium Proceedings.

[B48] Healthcare Information and Management Systems Society (HIMSS) (2004). 2004 National Health Information Infrastructure Survey. http://www.himss.org/content/files/2004HealthInfoInfrastructureSurvey.pdf.

[B49] Institute for Alternative Futures Health Information Systems Background Report. http://www.altfutures.com/BFP/Health_Information_Systems_2015.pdf.

[B50] Fairlie RW (2006). Race and the Digital Divide: Joint Center for Poverty Research Working Paper. http://www.eric.ed.gov/ERICDocs/data/ericdocs2sql/content_storage_01/0000019b/80/1a/63/33.pdf.

[B51] Gustafson DH, McTavish FM, Stengle W, Ballard D, Hawkins R, Shaw BR, Jones E, Julesberg K, McDowell H, Chen WC, Volrathongchai K, Landucci G (2005). Use and impact of ehealth systems by low-income women with breast cancer. J Health Commun.

[B52] Nielsen-Bohlman L, Panzer AM, Kindig, DA, Institute of Medicine. Committee on Health Literacy (2004). Health Literacy: A Prescription to End Confusion.

[B53] Moiduddin A, Gaylin DS Health Information Technology Adoption Among Health Centers: A Digital Divide in the Making?. National Health Policy Forum Background Paper.

[B54] Accenture Majority of Consumers Believe Electronic Medical Records Can Improve Medical Care, Accenture Survey Finds. http://newsroom.accenture.com/article_print.cfm?article_id=4236.

[B55] Accenture Consumers See Electronic Health Records as Important Factor When Choosing a Physician and Are Willing to Pay for the Service, Accenture Research Finds. http://newsroom.accenture.com/article_display.cfm?article_id=4509.

[B56] Thornewill J, Baluch J (2007). It takes a Whole Community of Caring to Improve Healthcare Quality and Contain Rising Health Costs. Greater Louisville eHealth Research Report.

[B57] Gearon CJ (2007). Perspectives on the Future of Personal Health Records. California. HealthCare Foundation.

[B58] Markle Foundation. Connecting Consumers (2006). Common Framework for Networked Personal Health Information. http://www.connectingforhealth.org/commonframework/docs/P9_NetworkedPHRs.pdf.

[B59] American National Standards Institute Healthcare Information Technology Standards Panel. Undated.

[B60] Health Level Seven, Inc HL7 Continuity of Care Document, a Healthcare IT Interoperability Standard, is Approved by Balloting Process and Endorsed by Healthcare IT Standards Panel. http://www.hl7.org/documentcenter/public/pressreleases/20070212.pdf.

[B61] Health Level Seven, Inc Health Level Seven's Personal Health Record Functional Model Approved as a Draft Standard for Trial Use. http://www.hl7.org/documentcenter/public/pressreleases/HL7_PRESS_20071205.pdf.

[B62] Adobe Adobe to Release PDF for Industry Standardization. http://www.adobe.com/de/aboutadobe/pressroom/pr/jan2007/OpenPDF.pdf.

[B63] Centers for Medicare and Medicaid Services Medicare Testing Personal Health Records to Help Beneficiaries Better Manage Own Health Care. http://www.cms.hhs.gov/apps/media/press/release.asp?Counter=2217.

[B64] Dossia Major U.S. Employers Join to Provide Lifelong Personal Health Records for Employees. http://www.dossia.org/consumers/.

[B65] Cowley S Microsoft beats Google to consumer health market. ChannelWeb.

[B66] Shabo A (2006). A Global socio-economic-medico-legal Model for the Sustainability of Longitudinal Electronic Health Records. Part 1 & 2. Methods Inf Med.

[B67] Gold JD, Ball MJ (2007). The Health Record Banking Imperative: A Conceptual Model. IBM Systems J.

[B68] Protti D (2007). Moving toward a Single Comprehensive Electronic Health Record for Every Citizen in Andalucía, Spain. Electronic Healthcare.

[B69] NHS National Services Scotland Emergency Care Summary marks 1 million patient consultations and national award commendations. http://www.nhsnss.org/supplementary_pages/news_detail.php?newsid=60.

[B70] eHealthNews.eu The Danish eHealth experience: One Portal for Citizens and Professionals. http://www.ehealthnews.eu/content/view/1041/62/.

[B71] Kane B, Sands DZ (1998). Guidelines for the clinical use of electronic mail with patients. The AMIA Internet Working Group, Task Force on Guidelines for the Use of Clinic-Patient Electronic Mail. J Am Med Inform Assoc.

[B72] http://www.projecthealthdesign.org/media/file/Common_Platform_Requirements.pdf.

[B73] Civan A, Skeels MM, Stolyar A, Pratt W (2006). Personal health information management: consumers' perspectives. AMIA Annu Symp Proc.

[B74] Markle Foundation. Connecting for Health (2008). Americans Overwhelmingly Believe Electronic Personal Health Records Could Improve Their Health. http://www.connectingforhealth.org/resources/ResearchBrief-200806.pdf.

[B75] Markle Foundation: Personal Health Working Group Final Report. http://www.connectingforhealth.org/resources/final_phwg_report1.pdf.

[B76] Robert Wood Johnson Foundation Expert Teams to Design New Solutions for Personal Health Records to Help Consumers Manage Their Health. http://www.rwjf.org/newsroom/newsreleasesdetail.jsp?productid=21905.

[B77] Privacilla.org Privacy Survey Design is Often Flawed. http://www.privacilla.org/fundamentals/surveyqs.html.

